# Immunological profiling of molecularly classified high-risk endometrial cancers identifies *POLE*-mutant and microsatellite unstable carcinomas as candidates for checkpoint inhibition

**DOI:** 10.1080/2162402X.2016.1264565

**Published:** 2016-12-09

**Authors:** Florine A. Eggink, Inge C. Van Gool, Alexandra Leary, Pamela M. Pollock, Emma J. Crosbie, Linda Mileshkin, Ekaterina S. Jordanova, Julien Adam, Luke Freeman-Mills, David N. Church, Carien L. Creutzberg, Marco De Bruyn, Hans W. Nijman, Tjalling Bosse

**Affiliations:** a Department of Obstetrics and Gynecology, University of Groningen, University Medical Center Groningen, Groningen, the Netherlands; b Department of Pathology, Leiden University Medical Center, Leiden, the Netherlands; c Department of Medical Oncology, INSERM U981, Gustave Roussy Cancer Center, Villejuif, France; d Queensland University of Technology (QUT), Translational Research Institute, Brisbane, QLD, Australia; e Institute of Cancer Sciences, University of Manchester, St Marys Hospital, Manchester, UK; f Division of Cancer Medicine, Peter MacCallum Cancer Centre, East Melbourne, VIC, Australia; g Center for Gynecological Oncology Amsterdam, VU Medical Center, Amsterdam, the Netherlands; h Tumour Genomics and Immunology Group, Oxford Centre for Cancer Gene Research, The Wellcome Trust Centre for Human Genetics, University of Oxford, Oxford, UK; i Oxford Cancer Centre, Churchill Hospital, Oxford, UK; j Department of Clinical Oncology, Leiden University Medical Center, Leiden, the Netherlands

**Keywords:** Checkpoint inhibition, endometrial cancer, high-risk, molecular classification, tumor-infiltrating lymphocytes

## Abstract

High-risk endometrial cancer (EC) is an aggressive disease for which new therapeutic options are needed. Aims of this study were to validate the enhanced immune response in highly mutated ECs and to explore immune profiles in other EC subgroups. We evaluated immune infiltration in 116 high-risk ECs from the TransPORTEC consortium, previously classified into four molecular subtypes: (i) ultramutated *POLE* exonuclease domain-mutant ECs (*POLE*-mutant); (ii) hypermutated microsatellite unstable (MSI); (iii) p53-mutant; and (iv) no specific molecular profile (NSMP). Within The Cancer Genome Atlas (TCGA) EC cohort, significantly higher numbers of predicted neoantigens were demonstrated in *POLE*-mutant and MSI tumors compared with NSMP and p53-mutants. This was reflected by enhanced immune expression and infiltration in *POLE*-mutant and MSI tumors in both the TCGA cohort (mRNA expression) and the TransPORTEC cohort (immunohistochemistry) with high infiltration of CD8^+^ (90% and 69%), PD-1^+^ (73% and 69%) and PD-L1^+^ immune cells (100% and 71%). Notably, a subset of p53-mutant and NSMP cancers was characterized by signs of an antitumor immune response (43% and 31% of tumors with high infiltration of CD8^+^ cells, respectively), despite a low number of predicted neoantigens. In conclusion, the presence of enhanced immune infiltration, particularly high numbers of PD-1 and PD-L1 positive cells, in highly mutated, neoantigen-rich *POLE*-mutant and MSI endometrial tumors suggests sensitivity to immune checkpoint inhibitors.

## Introduction

The development of novel immunotherapeutic strategies such as checkpoint inhibitors has the potential to transform the field of oncology. So far, durable responses have been established in subsets of patients, for example with metastatic melanoma, non-small cell lung cancer, and mismatch repair-deficient cancers including two patients with endometrial cancer (EC).^1-7^ Although the clinical efficacy of immune checkpoint inhibitors is evident in a subset of patients, selecting the patients who may benefit from this therapy remains challenging. A key mechanism for the benefit of immune checkpoint inhibition in these cancers is the induction of a strong neoantigen-driven T-cell response against the tumor. Indeed, comprehensive analysis of large genomic datasets such as The Cancer Genome Atlas (TCGA) have provided a clear link between mutational load and activation of the immune system, implicating the involvement of neoantigens in driving cytotoxic T-cell responses in cancer.^8-10^ Furthermore, several clinical trials have shown a strong association between the presence of high numbers of predicted neoantigens, immune infiltration and response to cancer immunotherapy.^11-15^ In particular, the presence of CD8^+^ cytotoxic T cells and expression of the immune checkpoints PD-1 and PD-L1 have been proposed as important predictors of objective tumor regression.^3,16^

Characterization of the immune contexture of individual tumors may provide guidance in selecting appropriate immunotherapy for each individual patient, especially when integrated with an analysis of genomic alterations.^10,17,18^ A molecular classification has recently been proposed by The Cancer Genome Atlas (TCGA), which identified four genomically distinct EC subgroups: an ultramutated group characterized by somatic mutations in the exonuclease domain of *POLE* (encoding the catalytic subunit of DNA polymerase epsilon), a microsatellite unstable (MSI) hypermutated group with many substitutions as well as insertions and deletions due to mismatch repair deficiency, a copy-number high (serous-like) group with frequent *TP53* mutation and a copy-number low (microsatellite stable (MSS)) group with no specific molecular profile (NSMP).^19^

In line with this, we, and others, have recently demonstrated high numbers of predicted immunogenic mutations and enhanced antitumor immune infiltration in ultramutated *POLE*-mutant and, to a lesser extent, in hypermutated microsatellite unstable EC.^20-23^ These studies combined with the emerging data linking mutational load, immune activation and response to cancer immunotherapy render *POLE*-mutated and MSI cancers plausible candidates for immune checkpoint inhibition.^3,10-13,24^ This is further underlined by recent case reports demonstrating the efficacy of anti-PD-1 inhibitors in advanced *POLE*-mutant or mismatch repair deficient cancers, including those of endometrial origin.^7,25,26^

In this study, we aimed to validate our previous findings of an enhanced immune response in *POLE*-mutant and MSI endometrial cancers in a cohort of high-risk patients. High-risk EC patients are a particularly relevant subgroup, as most have no or only very modest gain from standard local or systemic treatment after surgery. Novel treatment options are therefore urgently needed. The use of a molecularly defined cohort of high-risk endometrial cancer also enabled us to explore the immune profiles of the poorly characterized NMSP and p53-mutant subgroups. With this approach we provide a rationale for the administration of checkpoint inhibition strategies in subsets of *POLE*-mutant and MSI endometrial cancer patients.

## Results

### Enhanced infiltration of intratumoral CD3^+^, CD8^+^ and CD103^+^ lymphocytes in *POLE*-mutant and MSI tumors

**Figure 1.**
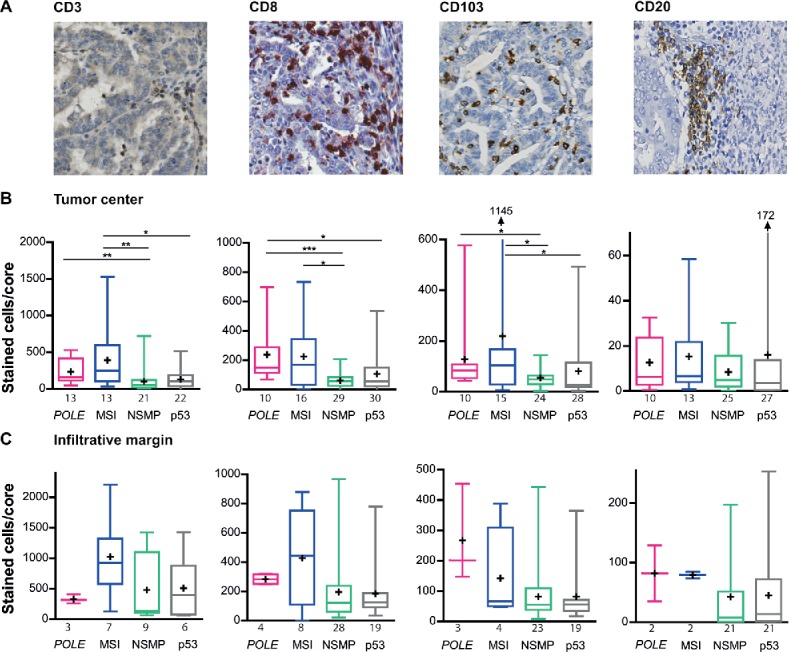
Infiltration of CD3^+^, CD8^+^, CD103^+^ and CD27^+^ cells in *POLE*-mutant, MSI, NSMP and p53-mutant endometrial cancers. (A) Representative immunohistochemical stainings of CD3^+^, CD8^+^, CD103^+^ and CD20^+^ cells. (B) Average number of positively stained intratumoral cells for each of the markers in the above panel, counted per core, corrected for the number of cells present. (C) Average number of positively stained cells for each of the markers in the above panel, counted per core within the infiltrative margin, corrected for the number of cells present. The numbers of cases analyzed for each molecular subgroup are listed below the *x*-axis. Boxes represent the interquartile range (IQR), with the upper whisker indicating the 75th percentile and the lower whisker the 25th percentile. The median and mean values are indicated by a horizontal line and cross, respectively. Abbreviations: *POLE, POLE*-mutant; MSI, microsatellite unstable; NSMP, no specific molecular profile; p53, p53-mutant. *p < 0.05, **p < 0.01, ***p < 0.001.

We first sought to characterize the lymphocytic infiltrate in the four EC molecular subtypes by immunohistochemical analysis of CD3^+^, CD8^+^, CD103^+^ and CD20^+^ (Fig. 1A and B). Compared to NSMP and p53-mutant tumors, both *POLE*-mutant and MSI tumors demonstrated increased density of CD3^+^ T-lymphocytes within the tumor center (*POLE* vs NSMP *p* = 0.002, MSI vs NSMP *p* = 0.001, MSI vs p53 *p* = 0.018). Staining for cytotoxic T-lymphocyte marker CD8^+^ and the intraepithelial T-lymphocyte marker CD103^+^ revealed similarly increased infiltrate in the tumor center (comparison of CD8^+^ cells: *POLE* vs NSMP *p* < 0.001; *POLE* vs p53 *p* = 0.021; MSI vs NSMP *p* = 0.016, comparison of CD103^+^ cells: *POLE* vs MSI *p* = 0.023; MSI vs NSMP *p* = 0.035; MSI vs p53 *p* = 0.030). Based on a median of 80.5 CD8^+^ cells/core in the whole cohort, 90% of *POLE*-mutant, 69% of MSI, 31% of NSMP and 43% of p53-mutant tumors were categorized as highly infiltrated with CD8^+^ cells. There was no difference in numbers of CD20^+^ B-lymphocytes within the tumor center. A combined analysis in which the two molecular subgroups with a high expected neoantigen load (*POLE*-mutant and MSI) were compared with the two molecular subgroups with lower expected neoantigen load (NSMP and p53-mutant), supported the apparent differences in immune infiltrate between EC subtypes (Fig. S1A).

Within the infiltrative margin, CD3^+^, CD8^+^, CD103^+^ or CD20^+^ infiltration did not significantly differ between the four molecular subgroups (Fig. 1C). Combined analysis showed a higher infiltration of CD8^+^ and CD103^+^ in *POLE*-mutant and MSI (CD8^+^ *p* = 0.010, CD103 *p* = 0.016, Fig. S1B).

### Increased infiltration of CD45RO^+^ and TIA-1^+^ lymphocytes in MSI tumors

**Figure 2.**
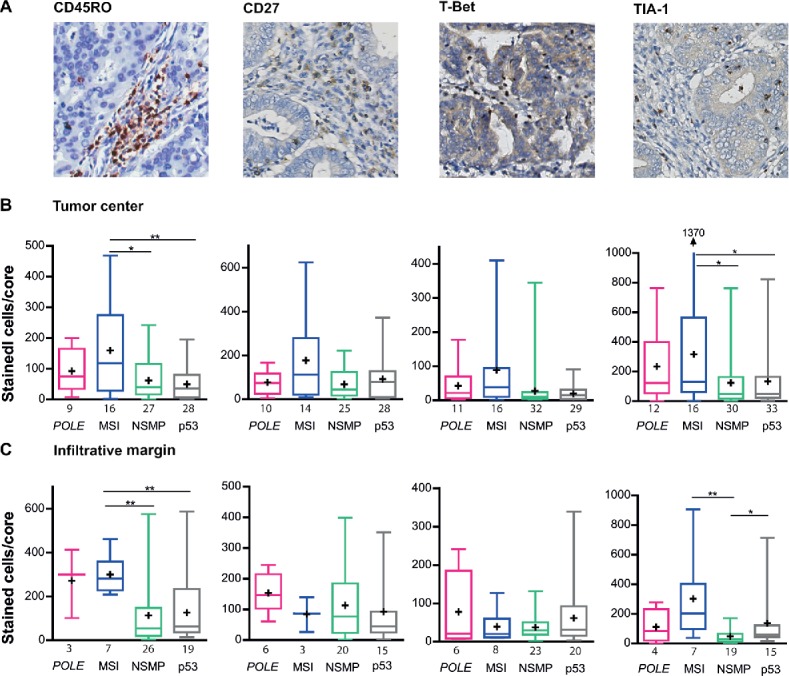
Infiltration of TIA-1^+^, T-Bet^+^, CD20^+^ and CD45RO^+^ cells in *POLE*-mutant, MSI, NSMP and p53-mutant endometrial cancers. (A) Representative immunohistochemical stainings of CD45RO^+^, CD27^+^, T-Bet^+^ and TIA-1^+^ cells. (B) Average number of positively stained intratumoral cells for each of the markers in the above panel, counted per core within the tumor center, corrected for the number of cells present. (C) Average number of positively stained cells for each of the markers in the above panel, counted per core within the infiltrative margin, corrected for the number of cells present. The numbers of cases analyzed for each molecular subgroup are listed below the *x*-axis. Boxes represent the interquartile range (IQR), with the upper whisker indicating the 75th percentile and the lower whisker the 25th percentile. The median and mean values are indicated by a horizontal line and cross, respectively. Abbreviations: *POLE, POLE*-mutant; MSI, microsatellite unstable; NSMP, no specific molecular profile; p53, p53-mutant. *p < 0.05, **p < 0.01, ***p < 0.001.

To analyze the function of the tumors' lymphocytic infiltrate, we performed immunohistochemistry for CD45RO, CD27, T-Bet and TIA-1 (Fig. 2A and B). Within the tumor center, MSI tumors contained more CD45RO^+^ memory T-lymphocytes compared with NSMP and p53-mutant tumors (MSI vs NSMP *p* = 0.029, MSI vs p53 *p* = 0.008). MSI tumors also harbored more TIA-1^+^ cytolytic lymphocytes within the tumor center (MSI vs NSMP *p* = 0.019, MSI vs p53 *p* = 0.043). There were no differences in the numbers of CD27^+^ naive T cells and T-Bet^+^ differentiated cells between the four molecular subgroups. Combined analysis of molecular groups revealed the presence of more CD45RO^+^ and TIA-1^+^ cells in *POLE*-mutant/MSI tumors compared with NSMP/p53-mutant tumors (Fig. S2A). Moreover, this also demonstrated higher numbers of T-Bet^+^ differentiated cells within *POLE*-mutant/MSI tumors compared with NSMP/p53-mutant tumors (*p* = 0.021).

Concordant with our findings in the tumor center, the infiltrative margin of MSI tumors contained more CD45RO^+^ lymphocytes (MSI vs NSMP *p* = 0.002, MSI vs p53 *p* = 0.003) and more TIA-1^+^ cytolytic T-lymphocytes (MSI vs NSMP *p* = 0.002, Fig. 2C). NSMP tumors demonstrated more TIA-1^+^ lymphocytes compared with p53-mutant tumors (NSMP vs p53 *p* = 0.023). The numbers of CD27^+^ and T-Bet^+^ cells did not significantly differ between the four molecular subgroups. Data from the combined analyses supported the increased density of CD45RO^+^ and TIA-1^+^ cells within *POLE*-mutant/MSI tumors (Fig. S2B).

### Increase in infiltration of PD-1^+^ and PD-L1^+^ lymphocytes in *POLE*-mutant and MSI tumors

**Figure 3.**
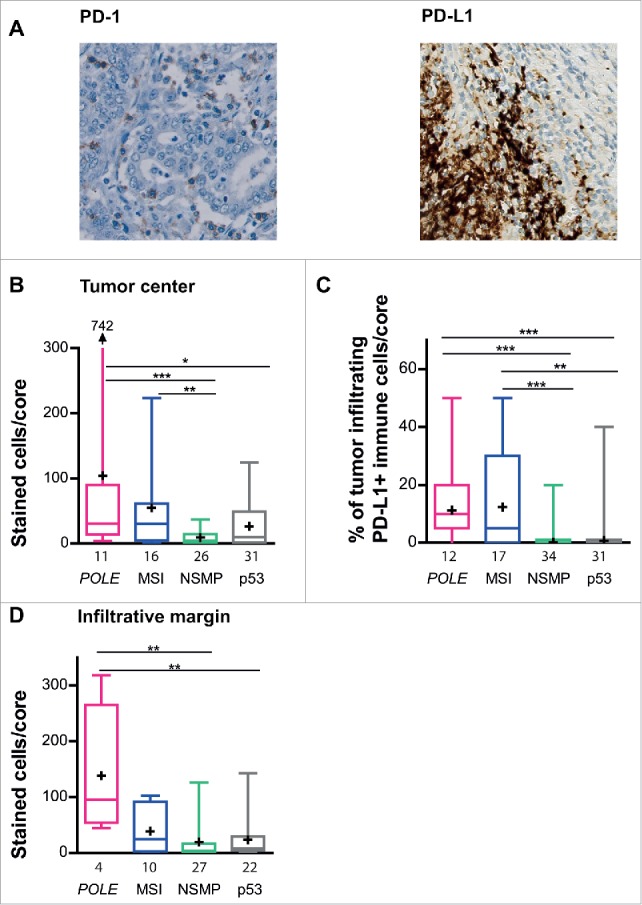
Infiltration of PD-1^+^ and PD-L1^+^ cells in *POLE*-mutant, MSI, NSMP and p53-mutant endometrial cancers. (A) Representative immunohistochemical stainings of PD-1^+^ and PD-L1^+^ cells. (B) Average number of PD1^+^ cells counted per core within the tumor center, corrected for the number of cells present. (C) Percentage of PD-L1^+^ tumor-infiltrating immune cells within the tumor core and infiltrative margin core. (D) Average number of PD1^+^ stained cells counted per core within the infiltrative margin. The numbers of cases analyzed for each molecular subgroup are listed below the x-axis. Boxes represent the interquartile range (IQR), with the upper whisker indicating the 75th percentile and the lower whisker the 25th percentile. The median and mean values are indicated by a horizontal line and cross, respectively. Abbreviations: *POLE, POLE*-mutant; MSI, microsatellite unstable; NSMP, no specific molecular profile; p53, p53-mutant. *p < 0.05, **p < 0.01, ***p < 0.001.

The increased lymphocytic infiltrate of *POLE*-mutant and MSI tumors, in combination with their expected ultramutated (*POLE*-mutant tumors) or hypermutated (MSI tumors) status, prompted us to investigate the presence of PD-1^+^ and PD-L1^+^ cells within this cohort (Fig. 3A).

The tumor center of *POLE*-mutant and MSI tumors harbored high numbers of PD-1^+^ immune cells (*POLE* vs NSMP *p* < 0.001, *POLE* vs p53 *p* = 0.050, and MSI vs NSMP *p* = 0.003, Fig. 3B). This was supported by the combined analysis (Fig. S3A). Based on a median of 14.0 PD-1^+^ cells/core in all patients, 73% of *POLE*-mutant, 69% of MSI, 31% of NSMP and 48% of p53-mutant tumors were categorized as highly infiltrated with PD-1^+^ cells.

*POLE*-mutant and MSI tumors showed markedly increased infiltration of PD-L1^+^ immune cells within the tumor center compare with NSMP and p53-mutant tumors (*POLE* vs NSMP *p* < 0.001, *POLE* vs p53 *p* < 0.001, MSI vs NSMP *p* < 0.001, MSI vs p53 *p* = 0.002, Fig. 3C). The combined analysis showed similar results (Fig. S3B). In total, 100% of *POLE*-mutant, 71% of MSI, 18% of NSMP and 29% of p53-mutant tumors were categorized as PD-L1^+^ (based on the immune score). Strikingly, only one tumor sample, a p53-mutant EC, contained PD-L1 expressing tumor cells (noted as a positive tumor score, data not shown).

Within the infiltrative margin, only the *POLE*-mutant subgroup showed high densities of PD-1^+^ immune cells (*POLE* vs NSMP *p* = 0.008, *POLE* vs p53 *p* = 0.007, Fig. 3D). Combined analysis supported the presence of high numbers of PD-1^+^ cells within the *POLE*-mutant/MSI group compare with the NSMP/p53-mutant group (Fig. S3C).

### PD-L1 is preferentially expressed on myeloid cells

**Figure 4.**
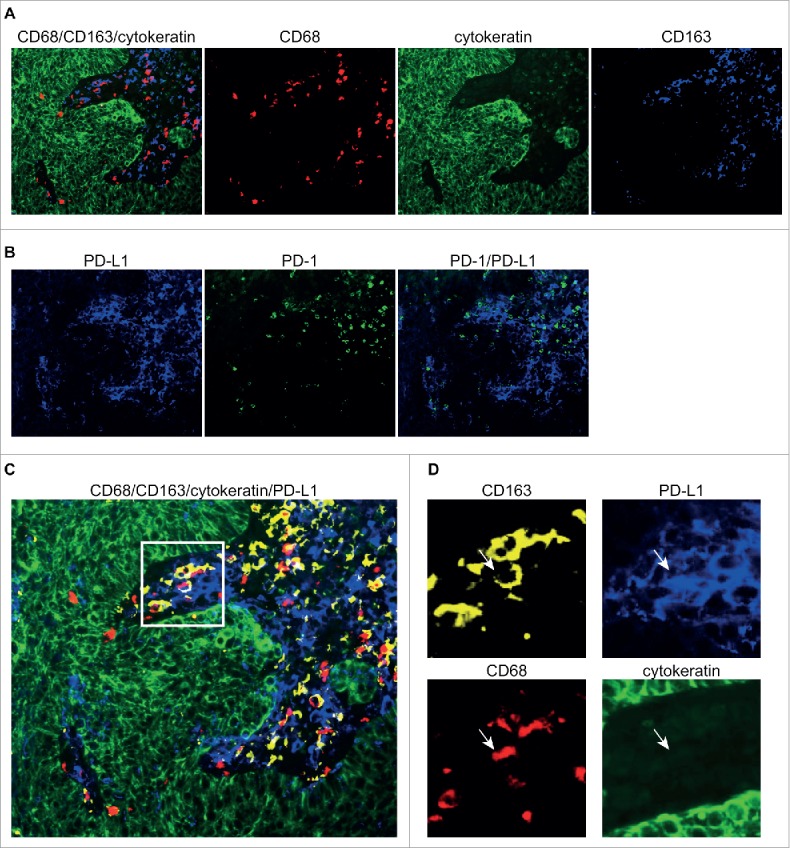
Immunofluorescent stainings of PD-1, PD-L1 and myeloid markers. Representative image of a *POLE*-mutant endometrial cancer stained with keratin (green)–CD163 (blue)–CD68 (red) in (A), and PD-1 (green)–PD-L1 (blue) in (B). The two triple immunofluorescent stainings from A and B, performed on sequentially cut slides, are layered in (C), with single channel markers for the inset in (D), with keratin (green), PD-L1 (blue), CD68 (red) and CD163 (yellow), demonstrating the co-localization of PD-L1 with myeloid markers.

Recently, several studies have shown PD-L1 expression on tumor-associated myeloid cells.^1,27-30^ Therefore, to determine whether this was also the case for our cohort, we performed two multi-color immunofluorescence stainings on consecutive whole slides of a highly infiltrated *POLE*-mutant tumor sample using the following combinations of monoclonal antibodies: CD68–CD163 – epithelial cell marker cytokeratin, and PD-L1–PD-1, respectively (Fig. 4). CD68^+^ and/or CD163^+^ myeloid cells (including macrophages and myeloid dendritic cells) were found in the stromal regions within the center of the tumor, demarcated by the cytokeratin^+^ tumor cells (Fig. 4A). PD-1^+^ and PD-L1^+^ cells were seen in close proximity, also predominantly located in the intratumoral stromal areas (Fig. 4B). A co-immunofluorescent staining of PD-1 and CD8 shows frequent co-localization, indicating that PD-1 can be expressed by (cytotoxic) T cells (Fig. S4). PD-L1 expression co-localized with CD68 and CD163, supporting the idea that in our cohort PD-L1 is not mainly expressed by tumor cells but by myeloid cells (Fig. 4C and D).

### TCGA RNA sequencing data demonstrates higher expression of CD8A, CD3E, ITGAE (CD103), MS4A1 (CD20), PTPRC (CD45RO), CD27,TBX21 (T-Bet) and PDCD1 (PD-1) in *POLE*-mutant and MSI tumors

**Figure 5.**
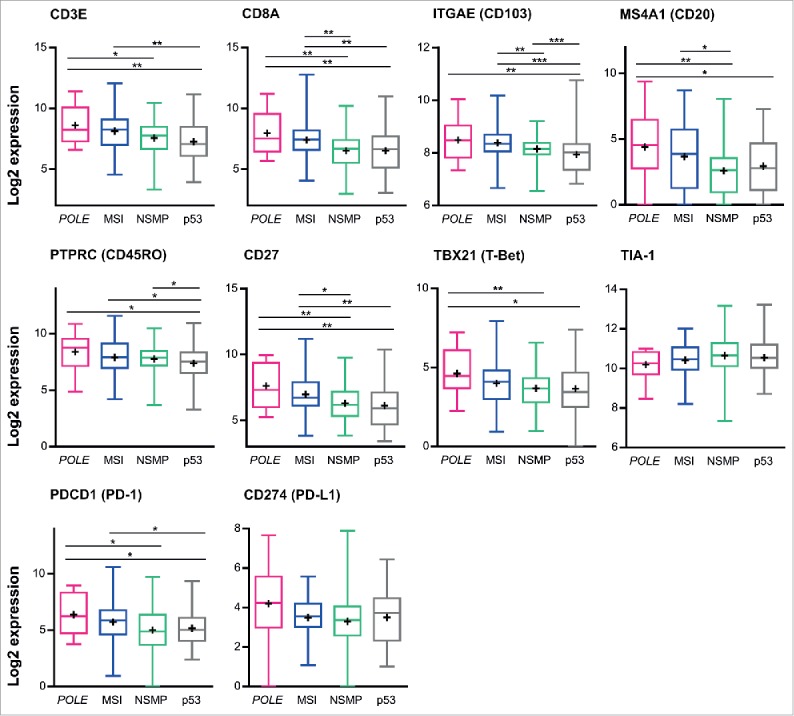
Expression of immune markers in according to tumor molecular subtype in TCGA series. RSEM normalized RNAseq data were log2 transformed and analyzed according to tumor molecular subtype. Boxes represent the interquartile range (IQR), with the upper whisker indicating the 75th percentile and the lower whisker the 25th percentile. The median and mean values are indicated by a horizontal line and cross, respectively. Abbreviations: *POLE, POLE*-mutant; MSI, microsatellite unstable; NSMP, no specific molecular profile; p53, p53-mutant. *p < 0.05, **p < 0.01, ***p < 0.001.

Next, we compared our data with the expression of above-described immune markers in The Cancer Genome Atlas (TCGA) cancer cohort, which was originally used to devise the molecular classification of EC (Fig. 5).^19^ Previously, we have shown higher expression of, among others, CD3E, CD8A, TBX21 (T-Bet) and PDCD1 (PD-1) in *POLE*-mutant compared with MSI and MSS tumors.^20^ We now extend this analysis to specifically compare the four proposed prognostic subgroups.^19^ Of the 244 informative samples, the TCGA cohort included 18 *POLE*-mutant, 69 MSI, 96 NSMP and 62 *TP53*-mutant. Analysis of the RNA sequencing data of this cohort demonstrated higher expression of CD8A, CD3E, ITGAE (CD103), MS4A1 (CD20), PTPRC (CD45RO), CD27,TBX21 (T-Bet) and PDCD1 (PD-1) in *POLE*-mutant and MSI tumors compare with NSMP and *TP53*-mutant. TIA-1 expression did not differ between the four molecular subgroups. *POLE*-mutant ECs showed a trend toward increased expression of CD274 (PD-L1) (*p* = 0.057).

### Patients with *POLE*-mutated and MSI tumors have higher numbers of predicted neoantigens, regardless of their immune infiltration status

**Figure 6.**
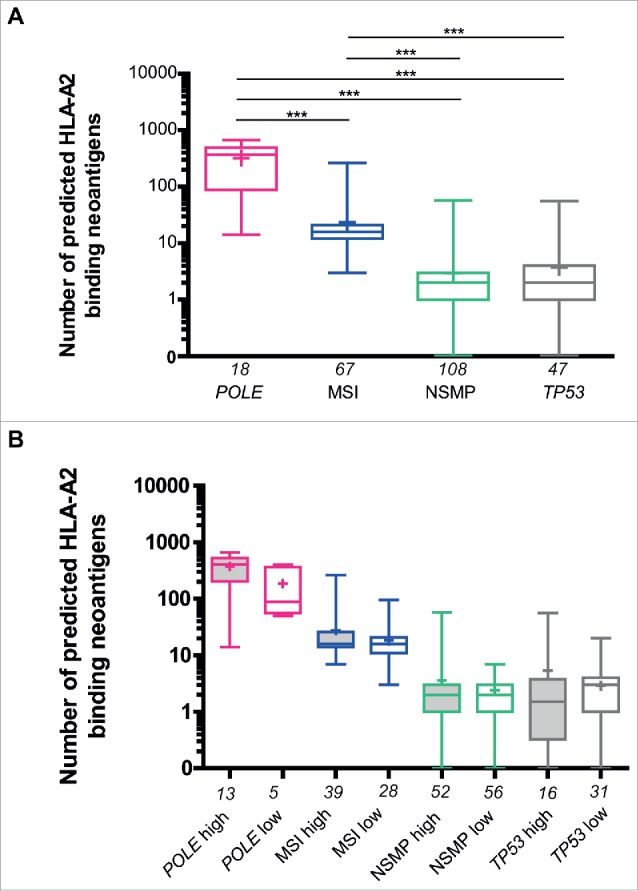
Predicted number of HLA-A2-binding neoantigens across the four molecular subgroups in The Cancer Genome Atlas endometrial cancer cohort. (A) Comparison between the number of predicted HLA-A2 binding neoantigens in *POLE*-mutant, MSI, NSMP and *TP53*-mutant subgroups based on RNAseq. (B) Comparison between patients with high and low infiltration (based on CD8A expression from RNAseq, relative to median within the group) of lymphocytes within *POLE*-mutant, MSI, NSMP and *TP53*-mutant subgroups. The numbers of cases analyzed for each molecular subgroup are listed below the x-axis. Boxes represent the interquartile range (IQR), with upper whisker indicating the 75th percentile and the lower whisker the 25th percentile. The median and mean values are indicated by a horizontal line and cross, respectively. Abbreviations: *POLE, POLE*-mutant; MSI, microsatellite unstable; NSMP, no specific molecular profile; p53, p53-mutant. *p < 0.05, **p < 0.01, ***p < 0.001.

The presence of a subset of *POLE*-mutant and MSI tumors with a relatively low immune infiltration and NSMP and *TP53*-mutant tumors with a relatively high immune infiltration led us to evaluate the relationship between immune infiltrate and numbers of predicted neoantigens within the TCGA cohort (Fig. 6). First of all, we demonstrated the presence of higher numbers of expected neoantigens in *POLE*-mutant and MSI tumors compared with NSMP and *TP53*-mutant tumors (Fig. 6A). The molecular subgroups were dichotomized according to CD8A expression from RNAseq data, with high infiltration defined as expression above the median of the respective molecular subgroup. Subsequently, we quantified predicted neoantigens for high and low infiltrated tumors within the molecular subgroups (Fig. 6B). No differences were found in the numbers of predicted neoantigens between samples with high or low CD8A expression within the molecular subgroups.

## Discussion

In this study we demonstrate the presence of high numbers of tumor-infiltrating T cells in *POLE*-mutant and MSI tumors, both predicted to be neoantigen-rich, from a clinically relevant cohort of high-risk EC patients. Moreover, these two molecular subtypes harbor high densities of PD-1- and PD-L1-expressing immune cells, rendering them attractive candidates for immune checkpoint inhibition strategies.

The presence of a prominent immune infiltrate in *POLE*-mutant and MSI high-risk EC is in concordance with our previous findings in a pre-selected cohort including 47 *POLE*-mutant, 49 microsatellite unstable and 54 microsatellite stable tumors, in which we demonstrated that *POLE*-mutant tumors, and to a lesser extent MSI tumors, are characterized by a robust intratumoral T-cell response.^20^ These initial findings have recently been extended to other unselected EC cohorts, in which high densities of peritumoral and tumor-infiltrating T-lymphocytes have been described in *POLE*-mutant tumors.^21,22,31^ High expression of PD-1 and PD-L1 on intraepithelial immune cells in *POLE*-mutant and MSI ECs has previously been suggested by Howitt et al, albeit in a cohort which included only three *POLE*-mutant cases.^21^ An interesting difference between the data presented by Howitt et al. and the present study is the expression of PD-L1 on tumor cells. Howitt et al. describe that 20% of ECs (*POLE*-mutant, MSI and MSS) show PD-L1^+^ tumor cells, whereas within our high-risk cohort only 1 out of 116 tumors showed any expression of PD-L1 on the tumor cells (using the same PD-L1 antibody). Our use of tissue microarrays may have led to an underestimation of PD-L1 expressing tumor cells, as PD-L1 expression is known to be heterogeneously distributed.^32^ Moreover, consecutive full slides of one *POLE*-mutant case were stained using multi-color immunofluorescence: PD-L1 expression was predominantly found in the intratumoral stromal regions in close proximity with PD-1^+^ cells. Furthermore, PD-L1 expression co-localized with CD68 and CD163, suggesting that in this case PD-L1 is primarily expressed by myeloid cells rather than tumor cells. PD-L1^+^ immune cells have previously been described by (among others) Heeren et al. and Herbst et al.; the latter also showed that PD-L1 positivity on immune cells, but not on tumor cells, was associated with response to immune checkpoint inhibition.^1,28^

Comparisons of outcomes from our immunohistochemical analyses in the TransPORTEC high-risk cohort and analyses of the RNA sequencing data from The Cancer Genome Atlas (TCGA) showed similar results for five out of ten markers, namely CD3, CD8^+^, CD103, CD45RO and PD1. The immunohistochemical analyses of the TransPORTEC cohort did not reveal significant differences in numbers of CD20^+^ and CD27^+^ cells between the four molecular subgroups, while analysis of the TCGA cohort demonstrated increased expression of CD20^+^ and CD27^+^ cells within the *POLE*-mutant and MSI subgroups. This inconsistency may be attributed to the use of a TMA for immunohistochemical analyses of CD20^+^ and CD27^+^ cells. These immune cells frequently reside in tertiary lymphoid structures in the myometrium, which are frequently seen in *POLE*-mutant tumors.^20,33-35^ The areas containing these structures may not have been present in the TMA. Second, outcomes regarding TIA-1, T-Bet and PD-L1 positivity were discordant. These differences may be due to the known discrepancy between mRNA and protein expression.^36^ Another possible explanation for these discrepancies may be the relatively high proportion of clear cell EC (15.5%) within the TransPORTEC high-risk cohort, while only endometrioid, serous and mixed histologies were included in the TCGA study.

The presence of high numbers of CD8^+^ and PD-1^+^ cells in *POLE*-mutant and MSI tumors may suggest the presence of high numbers of tumor-specific T cells targeting neoantigens within these subgroups of patients. Similarly, our analysis of the TCGA EC cohort demonstrates that *POLE*-mutant and MSI tumors are characterized by a significantly higher number of mutations predicted to result in major histocompatibility complex-binding neoantigens, and a correspondingly higher number of tumor-infiltrating CD8^+^ T cells, as assessed by CD8A mRNA levels. This link between neoantigen accumulation and infiltration by immune cells is supported by a recent genomic characterization of colorectal cancers, in which an association between high neoantigen load, overall lymphocytic infiltration, tumor-infiltrating lymphocytes and survival was demonstrated.^10^

Surprisingly, the number of predicted immunogenic mutations did not directly reflect the levels of CD8A mRNA expression within each molecular subgroup (Fig. 6). Similarly, in our immunohistochemical analysis, we found MSI tumors, expected to be neoantigen-rich, with almost no signs of CD8^+^ T-cell infiltration, and p53-mutant tumors, expected to have low numbers of neoantigens, with an enhanced intratumoral immune response. One explanation for this apparent discrepancy between immune infiltration and the number of predicted neoantigens could be that the nature (i.e., clonal vs subclonal) of the neoepitopes, instead of the crude number of predicted neoantigens, determine the degree of immune response.^13^ Another explanation may be that within our analyses only predicted binding to HLA-A*02:01 was taken into account rather than to individual HLA alleles. Furthermore, immune responses may be impeded by impairment of major histocompatibility complex (MHC) class I expression due to mutations in HLA, β-2 microglobulin and JAK-1 in highly mutated ECs.^20,37^ Therefore, a logical next step in understanding the interaction between neoepitopes and immune response within the four molecular subgroups would be the direct identification and characterization of tumor-specific T cells targeting these neoantigens, as has recently been performed by Gros et al. in melanoma.^24^

With regard to the p53-mutant tumors with an enhanced antitumor immune response despite low expected neoantigen load, we hypothesize that this response may be aimed at self-antigens or cancer/testis antigens instead of neoepitopes. Taking into account their unfavorable survival outcomes, further investigation of the highly infiltrated p53-mutant subset will be of great interest as this may provide new insight in the selection of candidates for immune checkpoint therapies.

The data on mutational load, neoantigens and immune infiltration reported by us and others suggest that checkpoint inhibition may be a strategy of particular interest for treating advanced stage patients with *POLE*-mutant and MSI tumors. Recent case reports provide proof of principle by demonstrating the efficacy of anti-PD-1 inhibitors in a limited number of advanced stage *POLE*-mutant or mismatch repair deficient cancers.^7,25,26^ Moreover, a Phase II trial evaluating immune related objective responses to Pembrolizumab in patients with or without mismatch repair (MMR) deficiency, demonstrated objective responses in 40% of patients with MMR deficient colorectal cancer and 71% of patients with MMR deficient non-colorectal cancers (including two ECs). Contrastingly, no objective responses were observed in the MMR proficient colorectal cancers. Moreover, data from this study adds to the growing body of evidence suggesting that high numbers of somatic mutations (in this case due to MMR deficiency) and high numbers of predicted neoantigens play an important role in the sensitivity to checkpoint inhibition.^11-13,15^ Furthermore, an in-depth analysis of patients treated with anti-PD-1 therapy prioritized PD-L1 expression as being the most closely associated with objective tumor regression.^38^ Further analyses of non-responders may uncover other mutations affecting epitope presentation, T-cell infiltration and response to checkpoint inhibition.

From a clinical point of view, as checkpoint inhibitors are associated with significant costs and potential toxicities, it is essential to select individual patients that will benefit from these therapies. Patients with low/intermediate-risk disease carrying *POLE* mutations have an excellent prognosis under standard treatment, and therefore checkpoint inhibition is unlikely to be appropriate for this group.^19,39-41^ However, (although infrequently occurring) *POLE*-mutant and MSI patients with recurring or metastatic disease are possible candidates.^7,25,26^ Clinical trials, in which high-risk EC patients are grouped according to molecular subtype, will be required to determine clinical benefit of immunotherapy.

Importantly, the data thus far regarding *POLE*-mutant EC may be applicable to other tumor types harboring *POLE* mutations. While *POLE* mutations are found in 7–12% of EC, they are also found in other malignancies including colorectal cancers, cancers of the brain, breast, pancreas and stomach, albeit at lower frequencies.^19,39,42-48^ Although a prognostic advantage of this mutation has now been established in glioblastoma and stage II/III colorectal cancer, patients with recurrent or metastatic hypermutated disease may also benefit from immunotherapeutic strategies such as checkpoint inhibitors as proposed for EC.^7,47,49^ Basket trials stratifying patients according to tumor molecular alterations such as *POLE* mutations should be initiated to investigate whether these patients may also benefit from checkpoint inhibition.

In summary, taking into account the strong immune infiltration, high numbers of PD-1^+^ and PD-L1^+^ lymphocytes, large numbers of somatic mutations and neoantigens, and the recently demonstrated clinical efficacy in these cohorts of patients, *POLE*-mutant and MSI tumors are expected to benefit from checkpoint inhibition.^21,25,50^

## Methods

### Selection of patients and tissues

A previously described cohort of 116 high-risk EC patients was used in this study (Table 1).^42^ In brief, tumor tissues from high-risk EC patients were selected from partner institutions of the TransPORTEC consortium using inclusion criteria of the PORTEC-3 study. Patients included in the PORTEC-3 had EC with one of the following FIGO 2009 stages and grade: 1A grade 3 with myometrial and lymphovascular space invasion; IB grade 3; II, IIIA or IIIC; IIIB if only parametrial invasion; stage IA (with invasion), 1B, II or III with serous or clear cell histology.^51^

**Table 1.** Clinicopathological characteristics of the high-risk endometrial cancer patient cohort.

	All patients		*POLE*-mutant		MSI		NSMP		p53-mutant		
	*N* = 116		*N* = 15		*N* = 19		*N* = 42		*N* = 40		
	*N*	%	*N*	%	*N*	%	*N*	%	*N*	%	*p*-value
Age at diagnosis (years)											
Mean (range)	66 (21–85)		61 (49–80)		65 (49–82)		64 (21–84)		71 (45–85)		0.004
Stage											
I	42	36.2	8	53.3	5	26.3	16	38.1	13	32.5	0.246
II	21	18.1	3	20.0	2	10.5	12	28.6	4	10.0	
III	41	35.3	3	20.0	10	52.6	11	26.2	17	42.5	
IV	11	9.5	1	6.7	2	10.5	3	7.1	5	12.5	
Unknown	1	0.9	0	0.0	0	0.0	0	0.0	1	2.5	
Tumor type											
Endometrioid	86	74.1	14	93.3	17	89.5	35	83.3	20	50.0	<0.001
Serous	12	10.3	0	0.0	0	0.0	0	0.0	12	30.0	
Clearcell	18	15.5	1	6.7	2	10.5	7	16.7	8	20.0	
Grade											
1	13	11.2	0	0.0	2	10.5	8	19.0	3	7.5	0.036
2	5	4.3	1	6.7	3	15.8	1	2.4	0	0.0	
3	98	84.5	14	93.3	14	73.7	33	78.6	37	92.5	
Lymphovascular space invasion											
Yes	55	47.4	6	40	15	78.9	18	42.9	16	40	0.103
No	40	34.5	9	60.0	2	10.5	18	42.9	11	27.5	
Unknown	21	18.1	0	0.0	2	10.5	6	14.3	13	32.5	
Depth of myometrial invasion											
<50%	23	19.8	4	26.7	2	10.5	6	14.3	11	27.5	0.261
>50%	87	75.0	11	73.3	17	89.5	33	78.6	26	65.0	
Unknown	6	5.2	0	0.0	0	0.0	3	7.1	3	7.5	
Adjuvant therapy											
Yes	82	70.7	14	93.3	15	78.9	33	78.6	20	50.0	0.134
No	10	8.6	1	6.7	1	5.3	2	4.8	6	15.0	
Unknown	24	20.7	0	0.0	3	15.8	7	16.7	14	35.0	

Characteristics are shown for the whole group, as well as for each of the molecular subgroups analyzed. Abbreviations: MSI, microsatellite unstable; NSMP, no specific molecular profile.

### Construction of tissue microarray

Morphologically representative paraffin-embedded tissue blocks containing at least 70% tumor cells were selected by two experienced gyneco-pathologists (VS and TB). The selected tumor blocks were used to construct (and validate) a Tissue Microarray (TMA) as previously described.^42^ One millimeter-sized tumor (center of the tumor) and tumor/stroma (invasive margin) cores of each tumor block were randomly distributed on the TMA in triplicate.

### Assessment of *POLE*, MSI, p53 and NSMP status

Classification of patients into the four molecular subgroups was performed as previously described.^42^ In brief, tumor DNA isolation was performed fully automated using the Tissue Preparation System (Siemens Healthcare Diagnostics).^52^ Bi-directional Sanger sequencing was used to screen exons 9, 13 and 14 of the *POLE* exonuclease domain for somatic mutations. Microsatellite instability and p53 mutational status were determined as previously described.^42,53^

### Immunohistochemistry

TMA sections were deparaffinized and rehydrated. Antigen retrieval was performed using 0.01M citrate buffer pH 6.0, and endogenous peroxidase activity was blocked. Slides were incubated overnight at room temperature (CD3, TIA-1, T-Bet and PD-1), for 1 h at room temperature (CD8^+^, CD20) or overnight at 4 °C (CD103) with primary antibodies against CD3 (1:100, clone PS-1, Diagnostic BioSystems), CD8^+^ (1:50, clone C8/144B, DAKO), CD20 (1:200, clone L26, DAKO), CD103 (1:200, Integrin αEβ7, Abcam), TIA-1 (1:200, clone 2G9A10F5, Beckman Coulter), T-bet (1:400 in 10% normal goat serum, sc-21003, Santa Cruz Biotechnology), PD-1 (1:200, AF1086, R&D), and PD-L1 (1 µg/mL, clone E1L3N, Cell Signaling Technology). Slides were incubated with BrightVision Poly-HRP (poly-HRP-GAM/R/R, DPV0110HRP, Immunologic; CD3, TIA-1, T-bet), a goat HRP-polymer kit (GHP516H, Biocare Medical; PD-1), anti-mouse secondary antibody (K4007, DAKO, CD8^+^, CD20) or anti-rabbit secondary antibody (K4011, DAKO, CD103) for 30 min. For CD103, a slightly different method using avidin/biotin blocking was used as described previously.^54^ PD-L1 staining was performed using the Ventrana Discovery Ultra Platform for automatic staining, detection was performed using the Discovery Amp-HQ kit (tyramide-based amplification). Antibody binding was visualized with 3,3′-diamino-benzidine-tetrahydrochloride (DAB) and haematoxylin counterstaining. Slides were dehydrated and mounted before digitalization (Ultra Fast Scanner 1.6 RA. Philips or ScanScope, Aperio technologies) and analysis.

### Quantification of IHC

Total numbers of CD3^+^, CD8^+^, CD103^+^, CD27^+^, TIA-1^+^, T-Bet^+^, CD20^+^, CD45RO^+^ and PD-1^+^ cell numbers were quantified per core. The percentage of tumor and stroma surface area within each core were estimated, and used to extrapolate cell counts to 100% surface area. Cores taken from the tumor center were included in the analysis if at least two out of the three cores contained >20% tumor. Cores from the infiltrative margin were included in the analysis if at least two out of the three cores contained >20% stroma and if there was tumor tissue present. Average cell counts per 100% surface area were recorded for the tumor center and infiltrative margin. Slides were counted manually by two individuals (FE and IG) that were blinded for other clinicopathological data. Inter-observer variation was evaluated by Spearman rank correlation (median *R*^2^ 0.935, range 0.682–0.988).

Quantification of PD-L1 was evaluated on tumor-infiltrating immune cells and tumor cells as previously described.^1^ In brief, the proportion of PD-L1 expressing tumor cells (tumor score) was noted as a percentage of the total number of tumor cells within that core. Due to very low expression of PD-L1 it was decided to consider any expression of PD-L1 on tumor cells as positive. Furthermore, the percentage of tumor-infiltrating immune cells (immune score) with moderate to strong PD-L1 expression was registered. Immune cells were defined positive when cells displayed clearly visible cytoplasmic and/or membranous staining. Patients were included in the analysis if at least two out of three cores were evaluated; the final score was based on the core with the highest PD-L1 expression. For the analyses of the immune score, PD-L1 positivity was defined as >1% (based on the median score in the cohort).

### Immunofluorescence

Three combinations of multi-color immunofluorescent stainings were performed as described previously.^55^ The first combination consisted of anti-CD163 (polyclonal rabbit, ab87099, Abcam), anti-CD68 (monoclonal mouse IgG2a, clone 514H12, ABDserotec) and anti-keratin (monoclonal mouse IgG1, clone AE1/AE3, MAB3412, Millipore). The second combination consisted of anti-PD-L1 (polyclonal rabbit, clone SP142, Roche) and anti-PD1 (monoclonal mouse IgG1, clone NAT105, Abcam), and the third consisted of anti-CD8^+^ (mouse monoclonal IgG2b, clone 4B11, Novo Castra) and anti-PD-1 (polyclonal goat, R&D Systems).

In short, after slides were deparaffinized and rehydrated, antigen retrieval was achieved by microwave oven treatment in a Tris–EDTA buffer at pH 9.0. Slides were incubated with the listed primary antibodies overnight. The following secondary Alexa Fluor labeled antibodies were used for the CD163–CD68–keratin and PD-L1–PD-1 combinations: 647 goat anti-rabbit, 546 goat anti-mouse IgG2a, and 488 goat anti-mouse IgG1 (all from Invitrogen, Life Technologies, Carlsbad, USA). Donkey anti-goat 488 and donkey anti-mouse IgG 647 were used for PD-1/CD8^+^ detection. The slides were counterstained with DAPI and coverslipped. Immunofluorescent images were acquired with an LSM700 confocal laser scanning microscope equipped with an LCI Plan-Neofluar 25×/0.8 Imm Korr DIC M27 objective (Zeiss, Göttingen, Germany). Double or triple positivity of cells in the center of the tumor as well as at the invasive margin was determined using LSM Image Browser (version 4.2.0.121, Zeiss). Images from the two triple immunofluorescent stainings were merged using Adobe Photoshop CS6.

### TCGA RNA sequencing

TCGA RNAseq analysis was performed as previously reported.^19,20^ Data were downloaded from FireBrowse on November 11, 2014 (http://firebrowse.org/?cohort=UCECanddownload_dialog=true). In total, 245 samples with RSEM normalized data were available for analysis.

### Prediction of antigenic neoepitopes

Prediction of antigenic neoepitopes was performed as previously reported.^20^ In brief, an algorithm was developed to estimate the immunogenicity of individual tumors in which the following considerations were taken into account: (i) to generate a functional neoepitope a missense mutation must be expressed; (ii) most functional neoepitopes are predicted to bind MHC class I molecules (IC50 < 500 nM) by NetMHCPan.^8,56,57^; (iii) the likelihood that a neoepitope is antigenic is reduced if the corresponding wild-type peptide also binds the MHC with similar affinity as T cells to the epitope may be centrally deleted or tolerized.^58^ Our strategy was similar to that reported by others.^8,13,57,59^ For each tumor all possible 9mers for every missense mutation in expressed genes (defined as non-zero reads from RNAseq) and the binding affinity of the mutant and corresponding wild-type peptide for HLA-A*02:01 were calculated using NetMHCPan 2.8.^56^ If several peptides had an IC50 <500 nM, the strongest binder was used for analysis. We defined antigenic mutations as neoepitopes predicted to bind MHC molecules (IC50 < 500 nM) for which the corresponding wild-type peptide was not predicted to bind MHC (IC50 > 500 nM).

### Statistical methods

Comparison between clinicopathological characteristics of the four molecular subgroups was made using Kruskal–Wallis followed by Mann–Whitney *U* (for age) and χ^2^ tests (for all other variables). Correlations between immunohistochemical stainings and the four molecular subgroups were evaluated using Kruskal–Wallis followed by Mann–Whitney *U* tests. The same method was used to evaluate correlations between RNA expression from the TCGA cohort of immune-related genes and the four molecular subgroups. Additionally, analyses were performed combining *POLE*-mutant and MSI samples vs NSMP and p53-mutant samples. All tests were performed two-sided. Significance was defined as a *p*-value of < 0.05. Statistical analyses were performed using IBM SPSS version 22 (SPSS, Inc., Chicago, USA) and GraphPad Prism (GraphPad Software, Inc., CA, USA).

## Supplementary Material

KONI_A_1264565_supplementary_data.zip

## References

[1] Herbst RS, Soria J-C, Kowanetz M, Fine GD, Hamid O, Gordon MS, Sosman JA, McDermott DF, Powderly JD, Gettinger SN et al. Predictive correlates of response to the anti-PD-L1 antibody MPDL3280A in cancer patients. Nature 2014; 515:563-7; PMID:25428504; http://dx.doi.org/10.1038/nature14011 25428504 PMC4836193

[2] Hodi FS, O'Day SJ, McDermott DF, Weber RW, Sosman JA, Haanen JB, Gonzalez R, Robert C, Schadendorf D, Hassel JC et al. Improved survival with ipilimumab in patients with metastatic melanoma. N Engl J Med 2010; 363:711-23; PMID:20525992; http://dx.doi.org/10.1056/NEJMoa1003466 20525992 PMC3549297

[3] Le DT, Uram JN, Wang H, Bartlett BR, Kemberling H, Eyring AD, Skora AD, Luber BS, Azad NS, Laheru D et al. PD-1 blockade in tumors with mismatch-repair deficiency. N Engl J Med 2015; 372:2509-20; PMID:26028255; http://dx.doi.org/10.1056/NEJMoa1500596 26028255 PMC4481136

[4] Powles T, Eder JP, Fine GD, Braiteh FS, Loriot Y, Cruz C, Bellmunt J, Burris HA, Petrylak DP, Teng S et al. MPDL3280A (anti-PD-L1) treatment leads to clinical activity in metastatic bladder cancer. Nature 2014; 515:558-62; PMID:25428503; http://dx.doi.org/10.1038/nature13904 25428503

[5] Hamid O, Robert C, Daud A, Hodi FS, Hwu W-J, Kefford R, Wolchok JD, Hersey P, Joseph RW, Weber JS et al. Safety and tumor responses with lambrolizumab (anti-PD-1) in melanoma. N Engl J Med 2013; 369:134-44; PMID:23724846; http://dx.doi.org/10.1056/NEJMoa1305133 23724846 PMC4126516

[6] Borghaei H, Paz-Ares L, Horn L, Spigel DR, Steins M, Ready NE, Chow LQ, Vokes EE, Felip E, Holgado E et al. Nivolumab versus Docetaxel in Advanced Nonsquamous Non-Small-Cell Lung Cancer. N Engl J Med 2015; 373:1627-39; PMID:26412456; http://dx.doi.org/10.1056/NEJMoa1507643 26412456 PMC5705936

[7] Bouffet E, Larouche V, Campbell BB, Merico D, de Borja R, Aronson M, Durno C, Krueger J, Cabric V, Ramaswamy V et al. Immune checkpoint inhibition for hypermutant glioblastoma multiforme resulting from germline biallelic mismatch repair deficiency. J Clin Oncol 2016; 34:2206-11; PMID:27001570; http://dx.doi.org/10.1200/JCO.2016.66.6552 27001570

[8] Brown SD, Warren RL, Gibb EA, Martin SD, Spinelli JJ, Nelson BH, Holt RA Neo-antigens predicted by tumor genome meta-analysis correlate with increased patient survival. Genome Res 2014; 24:743-50; PMID:24782321; http://dx.doi.org/10.1101/gr.165985.113 24782321 PMC4009604

[9] Rooney MS, Shukla SA, Wu CJ, Getz G, Hacohen N Molecular and genetic properties of tumors associated with local immune cytolytic activity. Cell 2015; 160:48-61; PMID:25594174; http://dx.doi.org/10.1016/j.cell.2014.12.033 25594174 PMC4856474

[10] Giannakis M, Mu XJ, Shukla SA, Qian ZR, Cohen O, Nishihara R, Bahl S, Cao Y, Amin-Mansour A, Yamauchi M et al. Genomic correlates of immune-cell infiltrates in colorectal carcinoma. Cell Rep 2016; 15:857-65; http://dx.doi.org/10.1016/j.celrep.2016.03.075 PMC4850357 27149842

[11] Pardoll DM The blockade of immune checkpoints in cancer immunotherapy. Nat Rev Cancer 2012; 12:252-64; PMID:22437870; http://dx.doi.org/10.1038/nrc3239 22437870 PMC4856023

[12] Rizvi NA, Hellmann MD, Snyder A, Kvistborg P, Makarov V, Havel JJ, Lee W, Yuan J, Wong P, Ho TS et al. Mutational landscape determines sensitivity to PD-1 blockade in non-small cell lung cancer. Science 2015; 348:124-8; http://dx.doi.org/10.1126/science.aaa1348 25765070 PMC4993154

[13] Snyder A, Makarov V, Merghoub T, Yuan J, Zaretsky JM, Desrichard A, Walsh LA, Postow MA, Wong P, Ho TS et al. Genetic basis for clinical response to CTLA-4 blockade in melanoma. N Engl J Med 2014; 371:2189-99; PMID:25409260; http://dx.doi.org/10.1056/NEJMoa1406498 25409260 PMC4315319

[14] Chan TA, Wolchok JD, Snyder A Genetic basis for clinical response to CTLA-4 blockade in melanoma. N Engl J Med 2015; 373:1984-1984. PMID:26559592; http://dx.doi.org/10.1056/NEJMc1508163 26559592

[15] Zou W, Wolchok JD, Chen L PD-L1 (B7-H1) and PD-1 pathway blockade for cancer therapy: Mechanisms, response biomarkers, and combinations. Sci Transl Med 2016; 8:328rv4; PMID:26936508; http://dx.doi.org/10.1126/scitranslmed.aad7118 26936508 PMC4859220

[16] Tumeh P, Harview C, Yearley J, Al E PD-1 blockade induces responses by inhibiting adaptive immune resistance. Nature 2014; 515:568-71; PMID:25428505; http://dx.doi.org/10.1038/nature13954 25428505 PMC4246418

[17] Mlecnik B, Bindea G, Angell HK, Maby P, Angelova M, Tougeron D, Church SE, Lafontaine L, Fischer M, Fredriksen T et al. Integrative analyses of colorectal cancer show immunoscore is a stronger predictor of patient survival than microsatellite instability. Immunity 2016; 44:698-711; PMID:26982367; http://dx.doi.org/10.1016/j.immuni.2016.02.025 26982367

[18] Becht E, Giraldo NA, Germain C, de Reyniès A, Laurent-Puig P, Zucman-Rossi J, Dieu-Nosjean M-C, Sautès-Fridman C, Fridman WH Immune contexture, immunoscore, and malignant cell molecular subgroups for prognostic and theranostic classifications of cancers. 2016; 130:95-190; PMID:26923001; http://dx.doi.org/23636398 10.1016/bs.ai.2015.12.002 26923001

[19] Cancer Genome Atlas Research Network, Kandoth C, Schultz N, Cherniack AD, Akbani R, Liu Y, Shen H, Robertson AG, Pashtan I, Shen R, Benz CC et al. Integrated genomic characterization of endometrial carcinoma. Nature 2013; 497:67-73; PMID:23636398; http://dx.doi.org/10.1038/nature12113 23636398 PMC3704730

[20] van Gool IC, Eggink FA, Freeman-Mills L, Stelloo E, Marchi E, de Bruyn M, Palles C, Nout RA, de Kroon CD, Osse EM et al. POLE proofreading mutations elicit an anti-tumor immune response in endometrial cancer. Clin Cancer Res 2015; 21:3347-56; PMID:25878334; http://dx.doi.org/10.1158/1078-0432.CCR-15-0057 25878334 PMC4627582

[21] Howitt BE, Shukla SA, Sholl LM, Ritterhouse LL, Watkins JC, Rodig S, Stover E, Strickland KC, D'Andrea AD, Wu CJ et al. Association of polymerase e–mutated and microsatellite-instable endometrial cancers with neoantigen load, number of tumor-infiltrating lymphocytes, and expression of PD-1 and PD-L1. JAMA Oncol 2015; 1:1319–23; PMID:26181000; http://dx.doi.org/27141333 10.1001/jamaoncol.2015.2151 26181000

[22] Bellone S, Centritto F, Black J, Schwab C, English D, Cocco E, Lopez S, Bonazzoli E, Predolini F, Ferrari F et al. Polymerase ε (POLE) ultra-mutated tumors induce robust tumor-specific CD4+ T cell responses in endometrial cancer patients. Gynecol Oncol 2015; 138:11-7; PMID:25931171; http://dx.doi.org/27141333 10.1016/j.ygyno.2015.04.027 25931171 PMC4469551

[23] van Gool IC, Bosse T, Church DN POLE proofreading mutation, immune response and prognosis in endometrial cancer. Oncoimmunology 2016; 5:e1072675; PMID:27141333; http://dx.doi.org/10.1080/2162402X.2015.1072675 27141333 PMC4839358

[24] Gros A, Parkhurst MR, Tran E, Pasetto A, Robbins PF, Ilyas S, Prickett TD, Gartner JJ, Crystal JS, Roberts IM et al. Prospective identification of neoantigen-specific lymphocytes in the peripheral blood of melanoma patients. Nat Med 2016; 22:433-8; PMID:26901407; http://dx.doi.org/10.1038/nm.4051 26901407 PMC7446107

[25] Mehnert JM, Panda A, Zhong H, Hirshfield K, Damare S, Lane K, Sokol L, Stein MN, Rodriguez-rodriquez L, Kaufman HL et al. Immune activation and response to pembrolizumab in POLE -mutant endometrial cancer. JCI 2016; 126:1-7; PMID:27159395; http://dx.doi.org/12091876 10.1172/JCI84940 PMC4887167 27159395

[26] Santin AD, Bellone S, Buza N, Choi J, Schwartz PE, Schlessinger J, Lifton RP Regression of chemotherapy-resistant Polymerase epsilon (POLE) ultra-mutated and MSH6 hyper-mutated endometrial tumors with nivolumab. Clin Cancer Res 2016; 22:5682-5687; PMID:27486176; http://dx.doi.org/12091876 10.1158/1078-0432.CCR-16-1031 PMC5135588 27486176

[27] Dong H, Strome SE, Salomao DR, Tamura H, Hirano F, Flies DB, Roche PC, Lu J, Zhu G, Tamada K et al. Tumor-associated B7-H1 promotes T-cell apoptosis: a potential mechanism of immune evasion. Nat Med 2002; 8:793-800; PMID:12091876; http://dx.doi.org/10.1038/nm0902-1039c 12091876

[28] Heeren AM, Koster BD, Samuels S, Ferns DM, Chondronasiou D, Kenter GG, Jordanova ES, de Gruijl TD High and interrelated rates of PD-L1+CD14+ antigen-presenting cells and regulatory T cells mark the microenvironment of metastatic lymph nodes from patients with cervical cancer. Cancer Immunol Res 2015; 3:48-58; PMID:25361854; http://dx.doi.org/10.1158/2326-6066.CIR-14-0149 25361854

[29] Schultheis AM, Scheel AH, Ozretić L, George J, Thomas RK, Hagemann T, Zander T, Wolf J, Buettner R PD-L1 expression in small cell neuroendocrine carcinomas. Eur J Cancer 2015; 51:421-6; PMID:25582496; http://dx.doi.org/10.1016/j.ejca.2014.12.006 25582496

[30] Curiel TJ, Wei S, Dong H, Alvarez X, Cheng P, Mottram P, Krzysiek R, Knutson KL, Daniel B, Zimmermann MC et al. Blockade of B7-H1 improves myeloid dendritic cell–mediated antitumor immunity. Nat Med 2003; 9:562-7; PMID:12704383; http://dx.doi.org/10.1038/nm863 12704383

[31] Bakhsh S, Kinloch M, Hoang LN, Soslow RA, Köbel M, Lee CH, Mcalpine JN, Mcconechy MK, Gilks CB Histopathological features of endometrial carcinomas associated with POLE mutations: Implications for decisions about adjuvant therapy. Histopathology 2015; 68:916-24; PMID:26416160; http://dx.doi.org/10.1111/his.12878 26416160 PMC5650229

[32] McLaughlin J, Han G, Schalper KA, Carvajal-Hausdorf D, Pelekanou V, Rehman J, Velcheti V, Herbst R, LoRusso P, Rimm DL et al. Quantitative assessment of the heterogeneity of PD-L1 expression in non-small-cell lung cancer. JAMA Oncol 2016; 2:46; PMID:26562159; http://dx.doi.org/10.1001/jamaoncol.2015.3638 26562159 PMC4941982

[33] Dieu-Nosjean MC, Giraldo NA, Kaplon H, Germain C, Fridman WH, Sautès-Fridman C Tertiary lymphoid structures, drivers of the anti-tumor responses in human cancers. Immunol Rev 2016; 271:260-75; PMID:27088920; http://dx.doi.org/10.1111/imr.12405 27088920

[34] Germain C, Gnjatic S, Dieu-Nosjean MC Tertiary lymphoid structure-associated B cells are key players in anti-tumor immunity. Front Immun 2015; 6:1-14; PMID:25657648; http://dx.doi.org/10.3389/fimmu.2015.00067 25755654 PMC4337382

[35] Kroeger DR, Milne K, Nelson BH Tumor-infiltrating plasma cells are associated with tertiary lymphoid structures, cytolytic T-cell responses, and superior prognosis in ovarian cancer. Clin Cancer Res 2016; 22:3005-15; PMID:26763251; http://dx.doi.org/10.1158/1078-0432.CCR-15-2762 26763251

[36] Zhang H, Liu T, Zhang Z, Chan DW, Rodland KD, Zhang H, Liu T, Zhang Z, Payne SH, Zhang B et al. Integrated proteogenomic characterization of human high-grade serous ovarian cancer resource integrated proteogenomic characterization of human high-grade serous ovarian cancer Cell 2016; 166:755-65; PMID:27372738; http://dx.doi.org/24714771 10.1016/j.cell.2016.05.069 PMC4967013 27372738

[37] Stelloo E, Versluis MA, Nijman HW, de Bruyn M, Plat A, Osse EM, van Dijk RH, Nout RA, Creutzberg CL, de Bock GH et al. Microsatellite instability derived *JAK1* frameshift mutations are associated with tumor immune evasion in endometrioid endometrial cancer. Oncotarget 2016; 7:39885-39893; PMID:27213585; http://dx.doi.org/24714771 10.18632/oncotarget.9414 PMC5129978 27213585

[38] Taube JM, Klein A, Brahmer JR, Xu H, Pan X, Kim JH, Chen L, Pardoll DM, Topalian SL, Anders RA Association of PD-1, PD-1 ligands, and other features of the tumor immune microenvironment with response to anti-PD-1 therapy. Clin Cancer Res 2014; 20:5064-74; PMID:24714771; http://dx.doi.org/10.1158/1078-0432.CCR-13-3271 24714771 PMC4185001

[39] Billingsley CC, Cohn DE, Mutch DG, Hade EM, Goodfellow PJ Prognostic significance of POLE exonuclease domain mutations in high-grade endometrioid endometrial cancer on survival and recurrence. Int J Gynecol Cancer 2016; 26:933-8; PMID:26937754; http://dx.doi.org/25505230 10.1097/IGC.0000000000000681 PMC4874836 26937754

[40] Church DN, Stelloo E, Nout RA, Valtcheva N, Depreeuw J, ter Haar N, Noske A, Amant F, Tomlinson IP, Wild PJ et al. Prognostic significance of POLE proofreading mutations in endometrial cancer. JNCI J Natl Cancer Inst 2014; 107:dju402; PMID:25505230; http://dx.doi.org/10.1093/jnci/dju402 25505230 PMC4301706

[41] McConechy MK, Talhouk A, Leung S, Chiu D, Yang W, Senz J, Reha-Krantz LJ, Lee C-H, Huntsman DG, Gilks CB et al. Endometrial carcinomas with POLE exonuclease domain mutations have a favorable prognosis. Clin Cancer Res 2016; 22:2865-73; PMID:26763250; http://dx.doi.org/25720322 10.1158/1078-0432.CCR-15-2233 26763250

[42] Stelloo E, Bosse T, Nout RA, MacKay HJ, Church DN, Nijman HW, Leary A, Edmondson RJ, Powell ME, Crosbie EJ et al. Refining prognosis and identifying targetable pathways for high-risk endometrial cancer; a TransPORTEC initiative. Mod Pathol 2015; 28:836-44; PMID:25720322; http://dx.doi.org/10.1038/modpathol.2015.43 25720322

[43] Church DN, Briggs SEW, Palles C, Domingo E, Kearsey SJ, Grimes JM, Gorman M, Martin L, Howarth KM, Hodgson S V et al. DNA polymerase ε and δ exonuclease domain mutations in endometrial cancer. Hum Mol Genet 2013; 22:2820-8; PMID:23528559; http://dx.doi.org/10.1093/hmg/ddt131 23528559 PMC3690967

[44] The Cancer Genome Atlas Network Comprehensive molecular characterization of human colon and rectal cancer. Nature 2012; 487:330-7; PMID:22810696; http://dx.doi.org/23945592 10.1038/nature11252 PMC3401966 22810696

[45] Alexandrov LB, Nik-Zainal S, Wedge DC, Aparicio SA, Behjati S, Biankin A V, Bignell GR, Bolli N, Borg A, Børresen-Dale A-L et al. Signatures of mutational processes in human cancer. Nature 2013; 500:415-21; PMID:23945592; http://dx.doi.org/10.1038/nature12477 23945592 PMC3776390

[46] Shinbrot E, Henninger EE, Weinhold N, Covington KR, Göksenin AY, Schultz N, Chao H, Doddapaneni H, Muzny DM, Gibbs RA et al. Exonuclease mutations in DNA polymerase epsilon reveal replication strand specific mutation patterns and human origins of replication. Genome Res 2014; 24:1740-50; PMID:25228659; http://dx.doi.org/10.1101/gr.174789.114 25228659 PMC4216916

[47] Erson-Omay EZ, Çağlayan AO, Schultz N, Weinhold N, Omay SB, Özduman K, Köksal Y, Li J, Serin Harmancı A, Clark V et al. Somatic POLE mutations cause an ultramutated giant cell high-grade glioma subtype with better prognosis. Neuro Oncol 2015; 17:1356-64; PMID:25740784; http://dx.doi.org/10.1093/neuonc/nov027 25740784 PMC4578578

[48] Rayner E, van Gool IC, Palles C, Kearsey SE, Bosse T, Tomlinson I, Church DN A panoply of errors: polymerase proofreading domain mutations in cancer. Nat Rev Cancer 2016; 16:71-81; PMID:26822575; http://dx.doi.org/10.1038/nrc.2015.12 26822575

[49] Domingo E, Freeman-Mills L, Rayner E, Glaire M, Briggs S, Vermeulen L, Fessler E, Medema JP, Boot A, Morreau H et al. Somatic POLE proofreading domain mutation, immune response, and prognosis in colorectal cancer: a retrospective, pooled biomarker study. Lancet Gastroenterol Hepatol 2016; 1:207-16; http://dx.doi.org/10.1016/S2468-1253(16)30014-0 28404093

[50] Gargiulo P, Della Pepa C, Berardi S, Califano D, Scala S, Buonaguro L, Ciliberto G, Brauchli P, Pignata S Tumor genotype and immune microenvironment in POLE-ultramutated and MSI-hypermutated Endometrial Cancers: New candidates for checkpoint blockade immunotherapy? Cancer Treat Rev 2016; 48:61-8; PMID:27362548; http://dx.doi.org/10.1016/j.ctrv.2016.06.008 27362548

[51] De Boer SM, Powell ME, Mileshkin L, Katsaros D, Bessette P, Haie-meder C, Ottevanger PB, Ledermann JA, Khaw P, Colombo A et al. Toxicity and quality of life after adjuvant chemoradiotherapy versus radiotherapy alone for women with high-risk randomised, phase 3 trial. Lancet Oncol 2016; 17:1-13; PMID:26758748; http://dx.doi.org/10.1016/S1470-2045(15)00568-9 27397040

[52] van Eijk R, Stevens L, Morreau H, van Wezel T Assessment of a fully automated high-throughput DNA extraction method from formalin-fixed, paraffin-embedded tissue for KRAS, and BRAF somatic mutation analysis. Exp Mol Pathol 2013; 94:121-5; PMID:22750048; http://dx.doi.org/10.1016/j.yexmp.2012.06.004 22750048

[53] Van Gool IC, Stelloo E, Nout RA, Nijman HW, Edmondson RJ, Church DN, MacKay HJ, Leary A, Powell ME, Mileshkin L et al. Prognostic significance of L1CAM expression and its association with mutant p53 expression in high-risk endometrial cancer. Mod Pathol 2016; 29:174-81; PMID:26743472; http://dx.doi.org/10.1038/modpathol.2015.147 26743472

[54] Workel HH, Komdeur FL, Wouters MCA, Plat A, Klip HG, Eggink FA, Wisman GBA, Arts HJG, Oonk MHM, Mourits MJE et al. CD103 defines intraepithelial CD8+ PD1+ tumour-infiltrating lymphocytes of prognostic significance in endometrial adenocarcinoma. Eur J Cancer 2016; 60:1-11. Available from: http://www.ncbi.nlm.nih.gov/pubmed/27038842; PMID:27038842; http://dx.doi.org/10.1016/j.ejca.2016.02.026 27038842

[55] Jordanova ES, Gorter A, Ayachi O, Prins F, Durrant LG, Kenter GG, van der Burg SH, Fleuren GJ Human leukocyte antigen class I, MHC class I chain-related molecule A, and CD8+/regulatory T-cell ratio: which variable determines survival of cervical cancer patients? Clin Cancer Res 2008; 14:2028-35; PMID:18381941; http://dx.doi.org/10.1158/1078-0432.CCR-07-4554 18381941

[56] Nielsen M, Lundegaard C, Blicher T, Lamberth K, Harndahl M, Justesen S, Røder G, Peters B, Sette A, Lund O et al. NetMHCpan, a method for quantitative predictions of peptide binding to any HLA-A and -B locus protein of known sequence. PLoS One 2007; 2:e796; PMID:17726526; http://dx.doi.org/23243591 10.1371/journal.pone.0000796 17726526 PMC1949492

[57] Khalili JS, Hanson RW, Szallasi Z In silico prediction of tumor antigens derived from functional missense mutations of the cancer gene census. Oncoimmunology 2012; 1:1281-9; PMID:23243591; http://dx.doi.org/10.4161/onci.21511 23243591 PMC3518500

[58] Duan F, Duitama J, Al Seesi S, Ayres CM, Corcelli SA, Pawashe AP, Blanchard T, McMahon D, Sidney J, Sette A et al. Genomic and bioinformatic profiling of mutational neoepitopes reveals new rules to predict anticancer immunogenicity. J Exp Med 2014; 211:2231-48; PMID:25245761; http://dx.doi.org/10.1084/jem.20141308 25245761 PMC4203949

[59] van Rooij N, van Buuren MM, Philips D, Velds A, Toebes M, Heemskerk B, van Dijk LJA, Behjati S, Hilkmann H, El Atmioui D et al. Tumor exome analysis reveals neoantigen-specific T-cell reactivity in an ipilimumab-responsive melanoma. J Clin Oncol 2013; 31:e439-42; PMID:24043743; http://dx.doi.org/10.1200/JCO.2012.47.7521 24043743 PMC3836220

