# Supplementary material for: Immunological profiling of molecularly classified high-risk endometrial cancers identifies POLE-mutant and microsatellite unstable carcinomas as candidates for checkpoint inhibition
Source: Oncoimmunology. 2016 Dec 9;6(2):e1264565. doi: 10.1080/2162402X.2016.1264565 (PMC5353925; doi:10.1080/2162402X.2016.1264565)
Supplement: KONI_A_1264565_supplementary_data.zip [file koni-06-02-1264565-s001.zip › KONI_A_1264565_s02 legends.docx]

**Supplemental Fig 1: Infiltration of CD3+, CD8+, CD103+ and CD20+ cells in *POLE*-mutant/MSI compared to NSMP/p53-mutant endometrial cancers**. A, Average number of positively stained cells for each of the markers in the above panel, counted per core within the tumor center, corrected for the number of cells present. B, Average number of positively stained cells for each of the markers in the above panel, counted per core within the infiltrative margin, corrected for the number of cells present. The numbers of cases analyzed for each molecular subgroup are listed below the x-axis. Boxes represent the interquartile range (IQR), with the upper whisker indicating the 75th percentile and the lower whisker the 25th percentile. The median and mean values are indicated by a horizontal line and cross, respectively. Abbreviations: *POLE, POLE*-mutant; MSI, microsatellite unstable; NSMP, no specific molecular profile; p53, p53-mutant.* = p<0.05, **= p<0.01, ***= p<0.001.

**Supplemental Fig 2: Infiltration of CD45RO+, CD27+, T-Bet+ and TIA-1+ cells in *POLE*-mutant/MSI compared to NSMP/p53-mutant endometrial cancers**. A, Average number of positively stained cells for each of the markers in the above panel, counted per core within the tumor center, corrected for the number of cells present. B, Average number of positively stained cells for each of the markers in the above panel, counted per core within the infiltrative margin, corrected for the number of cells present. The numbers of cases analyzed for each molecular subgroup are listed below the x-axis. Boxes represent the interquartile range (IQR), with the upper whisker indicating the 75th percentile and the lower whisker the 25th percentile. The median and mean values are indicated by a horizontal line and cross, respectively. Abbreviations: *POLE, POLE*-mutant; MSI, microsatellite unstable; NSMP, no specific molecular profile; p53, p53-mutant.* = p<0.05, **= p<0.01, ***= p<0.001.

**Supplemental Fig 3: Infiltration of PD-1+ and PD-L1+ cells in *POLE*-mutant/MSI compared to NSMP/p53-mutant endometrial cancers.** A, Average number of PD1+ stained cells counted per core within the tumor center, corrected for the number of cells present. B, Percentage of tumor-infiltrating immune cells with moderate to strong PD-L1 expression per core within cores taken from the tumor and infiltrative margin, corrected for the number of cells present. C, Average number of PD1+ stained cells counted per core within the infiltrative margin, corrected for the number of cells present. The number of cases analyzed for each molecular subgroup are listed below the x-axis. Boxes represent the interquartile range (IQR), with the upper whisker indicating the 75th percentile and the lower whisker the 25th percentile. The median and mean values are indicated by a horizontal line and cross, respectively. Abbreviations: *POLE, POLE*-mutant; MSI, microsatellite unstable; NSMP, no specific molecular profile; p53, p53-mutant.* = p<0.05, **= p<0.01, ***= p<0.001.

**Supplemental Fig 4: Immunofluorescent staining of CD8 and PD-1.** Representative image of a *POLE-*mutant endometrial cancer stained with CD8 and PD-1, demonstrating co-localization of CD8 and PD-1 on immune cells.
